# Protocol for a clinical practice guideline on acupuncture for chronic non-specific low back pain

**DOI:** 10.3389/fmed.2026.1834549

**Published:** 2026-07-06

**Authors:** Jiacheng Zhang, Yusheng Li, Gaoxinli Liu, Shun Fan, Peizhen Zhang, Qiaoling Chen, Jing Ning, Huanan Li, Jingui Wang

**Affiliations:** 1 Tuina Department, First Teaching Hospital of Tianjin University of Traditional Chinese Medicine, Tianjin, China; 2 Tuina Department, National Clinical Research Center for Chinese Medicine, Tianjin, China; 3 Preventive Treatment of Disease Department, Guang’anmen Hospital, China Academy of Chinese Medical Sciences, Baoding, China; 4 Graduate School, Jiangxi University of Chinese Medicine, Nanchang, China

**Keywords:** acupuncture, chronic non-specific low back pain, guideline, protocol, treatment

## Abstract

**Background:**

Chronic non-specific low back pain (CNLBP) is a common musculoskeletal disease that troubles adults worldwide. Adverse reactions associated with drug therapy are unavoidable, so non-pharmacological treatments have gained attention. Acupuncture can effectively relieves pain, improve function, and avoid adverse drug reactions. However, existing guidelines for low back pain have a broad scope, and the evidence assessment of acupuncture for CNLBP is not comprehensive. In addition, there are various types of acupuncture, and there is still a lack of consensus on diverse acupuncture treatment protocols. Therefore, the formulation of this guideline will fill the gap and meet the clinical needs.

**Methods:**

The main steps for the formulation of this guideline will include: (1) Establishing a guideline development group; (2) Managing conflicts of interest; (3) Researching and identifying clinical questions; (4) Searching and assessing evidence; (5) Assessing and grading the quality of evidence; (6) Developing decision tables for recommendations; (7) Reaching consensus and making decisions on recommendations; (8) Formulating treatment protocols; (9) Drafting the guideline document; (10) Conducting peer review.

**Discussion:**

This guideline is the first to focus its scope on CNLBP. It systematically synthesizes research evidence on acupuncture treatment, combines experts’ clinical experience with patients’ preferences, and develops clinically applicable recommendations and treatment plans. We incorporated the GRADE approach for evidence grading and a Delphi process for expert consensus. This process enhanced the scientific rigor and practical applicability of the recommendations. This guideline aims to provide clinicians with standardized and operable acupuncture treatment plans, and promote the standardization of acupuncture in the management of CNLBP.

**Clinical trial registration:**

http://www.guidelines-registry.cn/, identifier [PREPARE-2024CN071].

## 1 Introduction

Low back pain (LBP) is the foremost musculoskeletal condition in adults, with some 90% of cases identified as non-specific low back pain (NLBP) (1). Chronic non-specific LBP (CNLBP) is defined as NLBP persisting for more than 12 weeks. In China, the annual prevalence of LBP in adults ranges from 20.88% to 29.88%, with a point prevalence of 6.11%–28.5% (2). Conversely, the 13.1% point prevalence of chronic LBP is observed in U.S. adults aged 20–69 years (3). In Africa, the point prevalence of LBP is 39%, with annual and lifetime prevalence rates of 57% and 47%, respectively—substantially higher than global estimates (4). Approximately two-thirds of NLBP patients may develop chronic symptoms (5). Moreover, the medical costs associated with CNLBP now exceed those of coronary heart disease, arthritis, diabetes, and cerebrovascular diseases, imposing a significant medical and economic burden on both patients and society (6). Therapeutic options for CNLBP include pharmacotherapy, physical therapy, exercise therapy, surgical treatment, acupuncture, tuina, traditional Chinese medicine, and gua sha (7). As an adjunct to conventional care, acupuncture offers a safe and effective approach to easing pain and reducing disability in adult CNLBP patients (8). Furthermore, it helps avoid adverse drug reactions, making it a valuable complementary therapy for this condition. In 2014, the China Association of Acupuncture and Moxibustion issued the inaugural Evidence-Based Clinical Practice Guideline for Acupuncture in the Management of LBP (9), which recommended various acupuncture approaches for acute and chronic LBP but did not distinguish between specific and non-specific types. Subsequently, in 2023, the Standardization Committee of the World Federation of Acupuncture-Moxibustion Societies issued the Clinical Practice Guideline for Acupuncture: NLBP (10). Although this guideline covers acute, subacute, and chronic stages of the condition, it addresses only three clinical questions related to CNLBP, with the included evidence limited to studies on filiform acupuncture and electroacupuncture. Notably, no consensus recommendation was formed regarding the optimal treatment frequency for CNLBP. In addition, multiple clinical guidelines recommend acupuncture for the management of LBP. A key distinction between CNLBP and specific low back pain is that CNLBP cannot be attributed to a single identifiable pathoanatomical cause. CNLBP also differs fundamentally from acute and subacute non-specific low back pain. The pain in acute and subacute cases is primarily driven by nociceptive stimulation from peripheral tissue injury, whereas the maintenance and amplification of pain in CNLBP involve central sensitization (11) and psychosocial factors (12), indicating that CNLBP has evolved into an independent chronic pain syndrome. Clinically, CNLBP is characterized by persistence and high recurrence rate, so its treatment strategy is significantly different from other low back pain subtypes (5). In addition to pain relief, treatment goals for CNLBP should include improving physical function, preventing recurrence, enhancing quality of life, reducing disability, and lowering healthcare utilization. Existing acupuncture guidelines cover only filiform acupuncture and electroacupuncture and have not reached a consensus on key parameters such as treatment frequency and course, which limits their ability to address the comprehensive treatment needs of CNLBP. Notably, an economic evaluation study showed that adding acupuncture to routine care for community-dwelling older adults with chronic low back pain not only significantly improved physical function but also reduced average annual total healthcare expenditure per person (13). This cost-effectiveness advantage is particularly important in the context of global population aging and the escalating economic burden of CNLBP. Traditional Chinese medicine has unique advantages in the prevention and treatment of chronic diseases. This guideline will focus specifically on CNLBP, synthesize research evidence to formulate recommendations, and provide standardized treatment protocols for multiple acupuncture techniques. It will clarify treatment procedures and key parameters to maximize the therapeutic benefits of acupuncture, aiming to provide standardized treatment plans for users and promote the standardized application of acupuncture for CNLBP. Owing to CNLBP’s characteristics of high prevalence, prolonged illness course and elevated recurrence risk, as well as the unique strengths of traditional Chinese medicine in the prevention and treatment of chronic disorders, this guideline is specifically tailored to CNLBP. It synthesizes the available research evidence on acupuncture for CNLBP to formulate recommendations and develop standardized treatment protocols. This is expected to provide clinicians and other guideline users with clear, evidence-based acupuncture protocols, thereby promoting the standardized application of acupuncture in the management of CNLBP.

## 2 Methods

**FIGURE 1:**
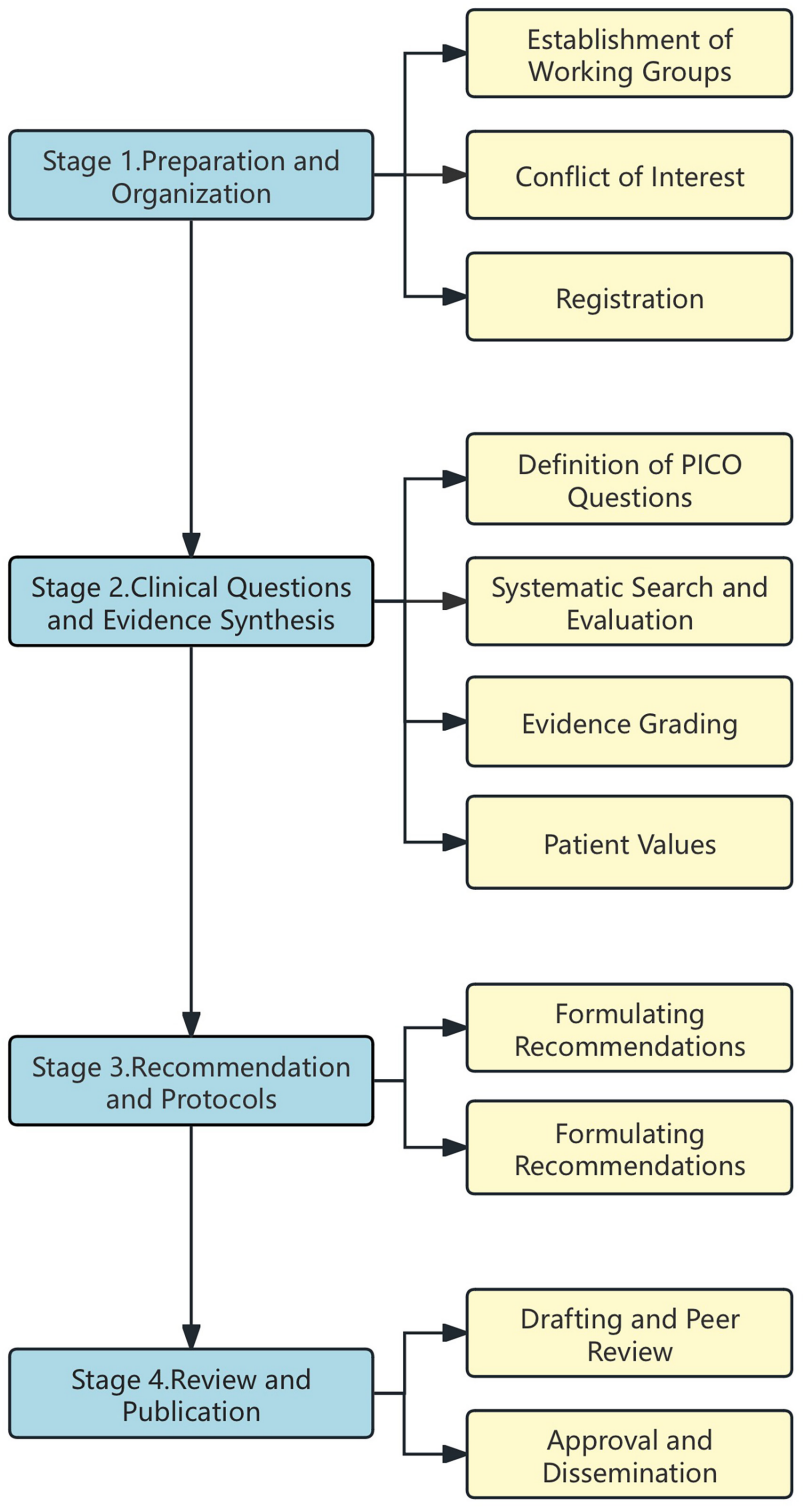
Flowchart of the guideline development process.

This guideline will be developed in accordance with the 2014 version of the WHO Handbook for Guideline Development (14). To assess the methodological quality of the included studies, tools such as RoB 2.0 and AMSTAR 2 will be used for evaluation. Evidence quality will be rated using the GRADE (Grading of Recommendations Assessment, Development and Evaluation) approach (15). The reporting and appraisal of this guideline will adhere to the RIGHT (Reporting Items for Practice Guidelines in Healthcare) standards (16) and the AGREE II (Appraisal of Guidelines for Research and Evaluation II) instrument (17). The detailed process steps can be found in Figure 1.

### 2.1 Initiating and support units

This guideline will be initiated by the First Teaching Hospital of Tianjin University of Traditional Chinese Medicine. The Evidence-Based and Medical Translation Center of Wuhan University will provide methodological support.

### 2.2 Trial registration

This guideline has been registered on the International Practice Guidelines Registry Platform. The registration number is PREPARE-2024CN071.

### 2.3 Target population for implementation of the guideline

Patients with CNLBP.

### 2.4 Guideline users

This guideline is intended for physicians who practice acupuncture, tuina, orthopedics and traumatology, or rehabilitation. Such clinicians specialize in traditional Chinese medicine or integrative Chinese-Western medicine and work across tiered hospitals and community health service facilities.

### 2.5 Guideline expert group

Six working groups will be established during the development of this guideline, including the Guideline Steering Committee, Guideline Consensus Expert Group, Secretariat Group, Evidence Evaluation Group, Treatment Protocol Development Group and External Review Expert Group (Supplementary Material 1). The responsibilities of the members of these six working groups are as follows.

#### 2.5.1 Guideline steering committee

The Guideline Steering Committee will consist of nine experts. Their main responsibilities are as follows: (1) Determine the theme and scope of the guideline; (2) Coordinate the work of other guideline working groups; (3) Manage conflicts of interest; (4) Review and approve the protocol; (5) Supervise the development process of the guideline; (6) Answer questions arising during the guideline development; (7) Approve and release the guideline.

#### 2.5.2 Guideline consensus expert group

The Guideline Consensus Expert Group will consist of 17 experts from the fields of clinical medicine and evidence-based methodology. The selection of these experts will fully consider regional and age factors. Their responsibilities are as follows: (1) Review and discuss clinical questions and outcome indicators, and vote on their importance; (2) Vote on the guideline recommendations and reach a consensus; (3) Review the treatment protocols developed by the Treatment Protocol Development Group; (4) They will also conduct a full review of the guideline text. In addition, the Guideline Consensus Expert Group will invite two patient representatives to vote on the importance of clinical questions and outcome indicators and to review the guideline manuscript. The expert group will provide them with professional interpretation of the guideline and invite them to provide comments and feedback on the clinical questions, outcome indicators, recommendations and treatment protocols. Selection and Engagement of Patient Representatives are detailed in Supplementary Material 3.

#### 2.5.3 Guideline secretarial group

Their main responsibilities are as follows: (1) Investigate and collect clinical questions and outcome indicators; (2) Extract treatment data from the included literature and organize Delphi surveys among the Treatment Protocol Development Group; (3) Analyze the Delphi survey results, summarize the treatment protocols that have reached consensus, and submit them to the Treatment Protocol Development Group and the Guideline Consensus Expert Group for review; (4) Organize consensus meetings on guideline recommendations; (5) Coordinate the work of other working groups; (6) Record the entire development process of the guideline; (7) Draft the first draft of the guideline; (8) Submit the guideline for publication.

#### 2.5.4 Guideline evidence evaluation group

This group will consist of evidence-based medicine experts from the units involved in the guideline development. Their main responsibilities are: (1) Search for, evaluate, and synthesize evidence, and assess the certainty of evidence; (2) Create summary of findings tables and recommendation decision tables.

#### 2.5.5 Treatment protocol development group

The Treatment Protocol Development Group will consist of 33 experts in acupuncture medicine. Its main responsibilities are as follows: (1) Review the candidate treatment protocol items and their corresponding evidence summaries provided by the Guideline Secretarial Group; (2) Independently score the parameter items of each treatment protocol, provide feedback, and reach consensus through the Delphi method; (3) Review and approve the final treatment protocols developed based on the Delphi survey results.

#### 2.5.6 Guideline external review group

This group will consist of acupuncture experts and experts in the methodology of guideline development who are not directly involved in the guideline formulation process. Their main responsibilities are: (1) Conduct a comprehensive review of the accuracy of the guideline; (2) Deliver specific feedback and actionable revision suggestions.

### 2.6 Conflict of interest management

To mitigate conflicts of interest, all members of the working groups will be required to complete a conflict of interest disclosure form, to state whether they have any potential financial, professional, or academic conflicts of interest.

### 2.7 Clinical questions

Secretariat Group will search relevant literature in Chinese and English databases using the search terms “acupuncture,” “CNLBP,” and “LBP” (Supplementary Material 2 for the search strategy). The Secretariat Group will formulate the collected questions into the PICO (Population, Intervention, Comparison, Outcomes) framework. Outcome indicators will be extracted and summarized from the literature, including randomized controlled trials (RCTs), systematic reviews (SRs), and guidelines related to CNLBP. Studies have shown that sham acupuncture is not an inert control measure (18). Therefore, the Secretariat Group will exclude studies using sham acupuncture as the sole control group when screening the literature. The Secretariat Group will invite experts from the fields of acupuncture and tuina medicine, rehabilitation medicine, traditional Chinese orthopedics and traumatology, and evidence-based methodology, as well as patient representatives, to participate in 2–3 rounds of consensus meetings on clinical questions and outcome indicators. Through these meetings, the priority ranking of clinical questions and outcome indicators for this guideline was determined. Patient representatives will participate in the scoring process for both clinical questions and outcome indicators to ensure that the perspectives of patients with CNLBP are fully reflected. Only outcome indicators rated as “very important” or “important” by both experts and patients will serve as the key basis for evidence synthesis and recommendation development.

Formulation of clinical questions: Clinical questions are scored on a scale of 1–5, where 1 indicates “very unimportant,” 2 indicates “unimportant,” 3 indicates “uncertain,” 4 indicates “important,” and 5 indicates “very important.” A score of 4 or higher defines a key clinical question that must be included in the guideline; a score of 3 defines an important clinical question that should be included; a score below 3 defines a minor clinical question that will not be considered for inclusion. If the mean score for a clinical question is 4 or higher and at least 75% of the scores are 4 or higher, consensus is considered reached, and the question should be included in the guideline. Conversely, if the mean score is 3 or lower and at least 75% of the scores are 3 or lower, the question should not be included.

The importance of outcome indicators is rated on a nine-point scale, where scores of 1–3 indicate “unimportant,” 4–6 indicate “important,” and 7–9 indicate “very important.” Based on the scoring results, outcome indicators are classified as follows: if the proportion of scores between 7 and 9 reaches 75% or more, the indicator is considered a key outcome indicator; if the proportion of scores between 4 and 6 reaches 75% or more, it is considered an important outcome indicator; and if the proportion of scores between 1 and 3 reaches 75% or more, it is considered a minor outcome indicator. For any clinical question or outcome indicator that fails to reach consensus after two rounds of voting, the final decision will be made through discussion by the members of the Guideline steering committee.

### 2.8 Evidence search and evaluation

Evidence search: Evidence retrieval will be conducted based on the PICO framework for each clinical question. The search terms will be developed around two main concepts: CNLBP and acupuncture, using a combination of MeSH terms and free-text words in both Chinese and English. A systematic search will be performed in eight Chinese and English databases, including China National Knowledge Infrastructure (CNKI), Wanfang Data Knowledge Service Platform (Wanfang), VIP Chinese Journal Database (VIP), Chinese Biomedical Literature Service System (SinoMed), PubMed, EMbase, Cochrane Library, and Web of Science. The search period will cover from the inception of each database to March 2026 and the literature types will be limited to RCTs and SRs/meta-analyses.

Evidence screening and data extraction: All identified literature records will be imported into EndNote X9 for centralized management. Duplicate entries will be eliminated via a combination of automatic software filtering and manual independent verification. Two reviewers will conduct independent screening of titles and abstracts for all retrieved records to identify potentially eligible studies. Thereafter, the full texts of these candidate studies will be retrieved, and the same two reviewers will perform separate assessments to determine their final inclusion. Any discrepancies arising during either screening phase will be resolved through joint discussion or by consulting a third independent reviewer. The final cohort of included studies will be confirmed upon the completion of this entire process.

Literature quality assessment: The quality of the included studies will be assessed using appropriate tools: RCTs will be evaluated with RoB 2.0,and SRs will be appraised using AMSTAR 2. Two reviewers will independently perform the quality assessment for each included study and subsequently cross-checked the results. Any disagreements during the assessment process will be resolved through discussion or by consultation with a third reviewer.

Systematic review development and updating: This guideline will only include SRs that meet the following criteria: (1) high methodological quality assessed by AMSTAR 2 (no critical flaws and no more than 1 non-critical flaw); (2) PICO elements fully consistent with the clinical questions of this guideline; (3) the search scope covers all databases specified in this guideline. If no eligible SR is identified, we will conduct a new systematic review. In addition, to ensure that included SRs include all recently published RCTs, we will perform a supplementary search for RCTs published after the search cutoff date of the SR. If no new RCTs are published after the cutoff date, we will directly use the results of that SR. If new RCTs are identified, we will assess their risk of bias using the RoB 2.0. We will then synthesize this new evidence to update the results of the original SR. We will then follow the decision framework proposed by Garner et al. (19). This framework will guide our evaluation. The evaluation will assess whether including the new RCTs is likely to meaningfully affect the review’s findings. The evaluation will consider three factors. First, whether the new RCTs change the direction or magnitude of the effect estimate. Second, whether the new RCTs alter the GRADE certainty rating for critical outcomes. Third, whether the new RCTs enable additional subgroup analyses or address previous limitations. If we judge that the new RCTs are likely to meaningfully affect the review’s findings, we will re-extract all primary data and conduct a new systematic review. Otherwise, we will retain the original systematic review results. To avoid double-counting of RCT data: RCTs already included in the high-quality SR will not be counted again. If the same RCT is included in multiple SRs, we will only extract data from the highest-quality SR. In case of data discrepancies, the results from the original RCT publication will be used as the reference.

Subgroup analysis: Acupuncture interventions are complex and may have significant heterogeneity. To explore the potential sources of this heterogeneity, members of the Guideline evidence evaluation Group will conduct subgroup analyses. The predefined subgroup analysis factors include acupuncture modality, treatment frequency, treatment course, and disease duration. Subgroup analyses will use interaction tests to compare effect sizes between subgroups. The analysis results will provide a basis for the formulation of recommendations and treatment protocols.

### 2.9 Grading of evidence quality

Evidence quality will be graded using the GRADE approach (Table 1). RCTs start as high-quality evidence but can be downgraded based on risk of bias, inconsistency, indirectness, imprecision, and publication bias. The final quality of evidence will be classified into four levels: high (A), moderate (B), low (C), and very low (D). As this guideline will not include observational studies, only RCTs will undergo quality grading. For each clinical question, the overall quality of the evidence body will be determined by the lowest quality rating among all key outcome indicators; in the absence of key outcome indicators, important outcome indicators will be considered instead. Members of the Guideline Evidence Evaluation Group will grade the quality of evidence separately for evidence bodies derived from different acupuncture modalities. The corresponding evidence level for each modality will also be presented in the summary of findings tables.

**TABLE 1:** Certainty of evidence and strength of recommendation grading criteria used in this guideline.

Classification	Explicit description
Certainty of evidence	
High (A)	We are very confident that the true effect lies close to that of the estimate of the effect.
Moderate (B)	We are moderately confident in the effect estimate. The true effect is likely to be close to the estimate of the effect, but there is a possibility that it is substantially different.
Low (C)	Our confidence in the effect estimate is limited. The true effect may be substantially different from the estimate of the effect.
Very low (D)	We have very little confidence in the effect estimate. The true effect is likely to be substantially different from the estimate of effect.
Recommended strength	
Strongly recommended/strongly not recommended	Clearly show that the intervention does more good than harm or more harm than good
Weakly recommended/weakly not recommended	Uncertainty about the benefits and disadvantages or evidence of equal quality of benefits and disadvantages

### 2.10 Formulation of recommendation opinions

This guideline will use the GRADE approach to classify the strength of recommendations into two levels: strong and weak. In formulating recommendations and determining their strength, a comprehensive assessment will be conducted based on evidence quality, the balance of benefits and harms, economic factors, and patients’ values and preferences, along with the expert consensus on treatment protocols obtained from the Delphi survey. The nominal group technique will be employed to determine recommendation strength. For each clinical question, the direction and strength of recommendations will be voted on using the GRADE grid (Table 2) (20). Consensus will be reached if either of the following criteria is met: (1) more than 50% of votes fall into any single grid, excluding the “not recommended temporarily” grid, allowing direct determination of both direction and strength; or (2) the combined votes from the two grids on either side of the “0” grid exceed 70%, establishing the recommendation direction with a “weak” strength designation. All other outcomes will be considered non-consensus. In cases where consensus is not achieved, the secretariat will revise the recommendations based on feedback from the guideline consensus group and organize subsequent voting rounds, with a maximum of three rounds. Recommendations reaching consensus will be reviewed and approved by the guideline steering committee. For controversial recommendations, the steering committee will be able to make revisions and improvements with the consent of at least two-thirds of the consensus group members; this process will be documented by the secretariat.

**TABLE 2:** The GRADE decision table for recommendations.

Strength of recommendation	Strong: “definitely do it”	Weak: “probably do it”	No explicit recommendation	Weak: “probably don’t do it”	Strong: “definitely don’t do it”
Definition	Desirable clearly outweighs undesirable	Desirable probably outweighs undesirable	Trade-offs equally balanced or uncertain	Undesirable probably outweighs desirable	Undesirable clearly outweighs desirable
Grade score of recommendations	2	1	0	−1	−2

### 2.11 Formulating the treatment protocols

To develop standardized acupuncture treatment protocols, this guideline will adopt the Delphi method to reach expert consensus on the basis of literature research. The items of the Delphi questionnaire will be jointly developed by frequency analysis (including acupoint correlation analysis) and subgroup analysis. The former will screen common treatment parameters and acupoint compatibility, while the latter, based on the frequency analysis, will verify the efficacy differences of different parameter combinations and assess their statistical significance.

Frequency analysis and acupoint correlation analysis: The Guideline Secretarial Group will extract treatment parameters from included RCTs and SRs. These parameters include acupuncture modality, acupoints or operation sites, needle specifications, manipulation techniques, electroacupuncture stimulation parameters, treatment frequency, single session duration, and total treatment course. All parameters will be classified and sorted by acupuncture modality. For acupuncture modalities with a sufficient number of studies, frequency analysis and acupoint correlation analysis will be conducted separately. We will summarize frequently occurring combinations of treatment parameters and patterns of acupoint compatibility, and develop preliminary candidate protocols for each acupuncture modality. For acupuncture modalities with a limited number of studies, only frequency analysis will be conducted, and separate preliminary candidate protocols will be developed directly.

Subgroup analysis: Acupuncture modality, treatment frequency, total treatment course, and disease duration are predefined as subgroup factors. Subgroup analysis will be used to test the efficacy differences of different parameter combinations and assess the statistical significance of these pooled effect sizes. Based on the above subgroup analysis results, the following criteria will be used to screen parameter combinations for each acupuncture modality: (1) Retention criteria: If the 95% confidence interval (95% CI) of the pooled effect size for a parameter combination does not include 0, and the effect direction favors acupuncture intervention, the combination is considered statistically significant and will be retained. If multiple subgroups within the same parameter category meet the retention criteria, combinations with larger pooled effect sizes and narrower confidence intervals will be prioritized for retention. If the *P*-value of the interaction test between subgroups is >0.05, all eligible combinations will be retained. (2) Exclusion criteria: If the 95% CI of the pooled effect size for a parameter combination includes 0, it is considered to have no clear evidence of efficacy and will be excluded from the subsequent Delphi survey. (3) Insufficient evidence: If fewer than two studies are available within a subgroup, subgroup analysis cannot be performed, and the corresponding parameter combination will proceed directly to the Delphi survey. All excluded parameter combinations will have their exclusion reasons noted in the summary of findings tables. After the above analysis, an optimized set of candidate items will be formed and will serve as the basis for the Delphi survey. The Delphi questionnaire will be classified by different acupuncture modalities, with separate evaluation items set for each modality.

Delphi method survey: The Guideline Secretarial Group will develop the questionnaire based on the optimized set of candidate items, adopt the Delphi method, and organize the 33 senior acupuncture and tuina experts from the Treatment Protocol Development Group to conduct the survey. These experts will evaluate and screen the items for each acupuncture modality one by one. The experts will rate each item using a five-point Likert scale (1 = strongly disagree, 2 = disagree, 3 = neutral, 4 = agree, 5 = strongly agree). A total of 2–3 rounds of this survey will be conducted. In each round, the Guideline Secretarial Group will distribute and collect the questionnaires, calculate the mean score, full-score ratio (proportion of scores = 5), consensus rate (proportion of scores ≥ 4), and coefficient of variation for each item, and feed the results back to the experts before initiating the next round. In addition, the Guideline Secretarial Group will calculate the expert response rate (positive coefficient) for each round of the Delphi survey to reflect the experts’ level of participation. Meanwhile, the expert authority coefficient (Cr) will be assessed in the questionnaire. A Cr value ≥ 0.7 is set as the acceptable threshold, indicating that the experts have a high level of authority and that the survey results are reliable.

Item retention criteria: Mean score ≥ 3.5, full-score ratio ≥ 0.2, consensus rate ≥ 0.6, and coefficient of variation ≤ 0.25. Items meeting all four criteria will be retained and will not enter subsequent rounds. Items meeting two or fewer criteria will be directly eliminated. Items meeting exactly three criteria will be reviewed by the Guideline Steering Committee to decide whether they should enter the next round. If an expert proposes a revision to an item and the revision is adopted by the Steering Committee, the revised item will enter the subsequent round and will be formally adopted if the results of that round meet the above retention criteria and more than half of the experts approve the revision. The finalized treatment protocols for each acupuncture modality will be compiled by the Guideline Secretarial Group and submitted to the Treatment Protocol Development Group and the Guideline Consensus Expert Group for review, serving as the basis for formulating the guideline recommendations.

### 2.12 Reporting and publishing the guideline

Guideline secretarial group will draft the first draft of the guideline following the AGREE II standards and submit it to the guideline consensus expert group for review and revision, resulting in a draft for public comment. This draft will then be reviewed by the external review expert group. Based on their feedback, the secretariat will revise and refine the guideline. The final version will be approved and released by the guideline steering committee.

### 2.13 Dissemination, implementation, and evaluation of the guideline

After its release, the guideline will be introduced and disseminated through academic conferences and social media platforms, such as WeChat official accounts and video channels of hospitals and relevant societies. Subsequently, multi-center, large-sample RCTs will be conducted to validate the treatment strategies recommended in the guideline and to evaluate its clinical applicability.

### 2.14 Updating the guideline

The guideline will be updated 2–3 years after its release. In practice, the timing of the update can be adjusted based on whether new, high-quality clinical evidence emerges and whether the update of the evidence has an impact on the recommendations of the guideline.

## 3 Discussion

Low back pain has become a global public health problem. It affects individuals of all ages. Most individuals experience LBP at some point in their lives (21). Acetaminophen and non-steroidal anti-inflammatory drugs (NSAIDs) are regarded as first-line interventions for the majority of patients (22). However, these drugs may cause gastrointestinal, hepatic, and renal damage, as well as an increased risk of cardiovascular events (23). Opioids are recommended as second-line treatment when NSAIDs are contraindicated, not tolerated, or ineffective (24). Opioid abuse and addiction pose significant risks. Therefore, non-pharmacological therapies are important in the management of CLBP. Multiple clinical guidelines recommend adding acupuncture to conventional treatment for patients with CLBP to relieve pain and improve physical function (25).

Acupuncture is based on the meridian theory of Traditional Chinese Medicine. It regulates the circulation of *qi* and blood in the lumbar region by stimulating specific acupoints. In addition, acupuncture can improve patient mood (26). The mechanism of acupuncture analgesia has been extensively studied. Acupuncture can induce signal changes in brain regions involved in the integration of pain signals, such as the insula, primary somatosensory cortex (S1 and S2), thalamus, and medial prefrontal cortex. It normalizes the functional connectivity between these regions and thalamic, cortical, and brainstem nuclei (27). Furthermore, acupuncture is found to trigger the release of endogenous opioid peptides within the central nervous system (CNS). This inhibits the ascending sensory pathway of pain and relieves a variety of pain symptoms (28). Studies have shown that electroacupuncture can block pain through peripheral, spinal, and supraspinal mechanisms (29). These mechanisms involve the activation of multiple bioactive substances, including opioids, serotonin, and norepinephrine.

Many previous studies have shown conflicting evidence on acupuncture for the treatment of CNLBP. This may be associated with the imprecision across acupuncture studies. However, acupuncture is still recognized as a promising therapy and can be recommended based on patients’ preferences, feasibility of implementation and treatment costs (28, 30). Therefore, how to evaluate the therapeutic effect of acupuncture, assist clinicians and patients in making appropriate judgments on the application scenarios of acupuncture, and provide effective, safe and standardized acupuncture treatment protocols constitutes the key difficulty in developing this guideline (31).

Based on this, the guideline development group carried out a literature review, supplemented it with the practical experience of authoritative experts, and fully considered a solid research foundation and clinicians’ recognition, thus making clinical issues better meet clinical needs. The GRADE approach served as the core framework for evidence assessment and recommendation formulation. Using the GRADE approach, the quality of included evidence was systematically graded based on multiple factors, including the magnitude of therapeutic effects, evidence quality, patient preferences and values, resource utilization, feasibility, equity, and the balance of benefits and harms, thereby ensuring the clinical applicability of the recommendations. Regarding the methodology, the guideline development group comprised experts from the fields of acupuncture and tuina, traditional Chinese medicine orthopedics, rehabilitation medicine, orthopedics, and evidence-based medicine. Each subgroup had clearly defined responsibilities, and the multidisciplinary composition of the group helped mitigate potential risks of bias, as well as academic and financial conflicts of interest during the evidence synthesis process.

Acupuncture is a complex intervention; therefore, the treatment protocols provided in this guideline should incorporate expert opinion and clinical experience to ensure their implementability (32). To this end, the guideline development group selected 33 clinicians based on regional and disciplinary representation, as well as literature review findings, to conduct a Delphi questionnaire survey aimed at formulating treatment protocols for various acupuncture approaches. This allows patients and clinicians to choose personalized treatments according to their individual preferences and available medical resources.

The development of this protocol reflects the high transparency of the guideline development process, which will encourage researchers to adopt rigorous and standardized methodologies in future guideline initiatives. Moving forward, this guideline will address unresolved clinical questions based on the latest evidence, thereby providing an evidence-based foundation for acupuncture treatment of chronic non-specific LBP.

The limitations of this study include: (1) This guideline only includes studies published in Chinese and English, which may overlook relevant studies in other languages and lead to incomplete evidence; (2) Only RCTs and SRs were included, while observational studies were excluded, which may limit the assessment of real-world effectiveness and long-term safety; (3) The experts involved in the development of this guideline were mainly from China, which may introduce geographical bias in the treatment protocols and consensus, thereby limiting the global applicability of the guideline.

## References

[1] Allegri M Montella S Salici F Valente A Marchesini M Compagnone C et al. Mechanisms of LBP: a guide for diagnosis and therapy. *F1000Res.* (2016) 5:F1000FacultyRev–1530. 10.12688/f1000research.8105.2 27408698 PMC4926733

[2] Chen D Chen CH Hu ZC Shao ZX Lin JL Wu AM. Prevalence of LBP in adult population in China: a systematic review. *Chin J Evid Based Med.* (2019) 19:651–5. 10.7507/1672-2531.201801044

[3] Shmagel A Foley R Ibrahim H. Epidemiology of chronic LBP in us adults: data from the 2009-2010 national health and nutrition examination survey. *Arthritis Care Res.* (2016) 68:1688–94. 10.1002/acr.22890 26991822 PMC5027174

[4] Morris LD Daniels KJ Ganguli B Louw QA. An update on the prevalence of LBP in Africa: a systematic review and meta-analyses. *BMC Musculoskelet Disord.* (2018) 19:196. 10.1186/s12891-018-2075-x 30037323 PMC6055346

[5] Itz CJ Geurts JW Van Kleef M Nelemans P. Clinical course of non-specific LBP: a systematic review of prospective cohort studies set in primary care. *Eur J Pain.* (2013) 17:5–15. 10.1002/j.1532-2149.2012.00170.x 22641374

[6] Deyo RA Dworkin SF Amtmann D Andersson G Borenstein D Carragee E et al. Report of the NIH task force on research standards for chronic LBP. *Pain Med.* (2014) 15:1249–67. 10.1111/pme.12538 25132307

[7] T Z D S Ah M. Recent clinical practice guidelines for the management of LBP: a global comparison. *BMC.* (2024) 25:344. 10.1186/s12891-024-07468-0 38693474 PMC11061926

[8] Asano H Plonka D Weeger J. Effectiveness of acupuncture for nonspecific chronic lbp: a systematic review and meta-analysis. *Med Acupunct.* (2022) 34:96–106. 10.1089/acu.2021.0057 35509875 PMC9057891

[9] Zhao H Liu BY Liu ZS Xie LM Fang YG Zhu Y et al. Clinical practice guidelines of using acupuncture for LBP. *World J Acupunct Moxibustion.* (2016) 26:1–14. 10.1016/S1003-5257(17)30016-8

[10] Liu XX Guo YY Fei YT Huang M Ye YM Yu JN et al. World federation of acupuncture-moxibustion societies clinical practice guideline on acupuncture-moxibustion: non-specific LBP recommendation summaries. *World J Acupunct-Moxibustion.* (2024) 34:213–21. 10.1016/J.WJAM.2024.06.005

[11] Woolf CJ. Central sensitization: implications for the diagnosis and treatment of pain. *Pain.* (2011) 152:S2–15. 10.1016/j.pain.2010.09.030 20961685 PMC3268359

[12] Gatchel RJ Peng YB Peters ML Fuchs PN Turk DC. The biopsychosocial approach to chronic pain: scientific advances and future directions. *Psychol Bull.* (2007) 133:581–624. 10.1037/0033-2909.133.4.581 17592957

[13] Herman PM Mann S DeBar LL Avins AL Justice M Nielsen A et al. Cost-effectiveness of acupuncture needling for older adults with chronic low back pain. *Spine.* (2026) 51:E65–75. 10.1097/BRS.0000000000005549 41493335 PMC12778958

[14] World Health Organization [WHO]. *WHO Handbook for Guideline Development 2nd ED.* Geneva: World Health Organisation (2014).

[15] Grade. *GRADE Handbook for Grading Quality of Evidence and Strength of Recommendations.* (2013). Available online at: www.guidelinedevelopment.org/handbook (accessed October 2013).

[16] Agree. *AGREEII User’s Manual and 23 Item Instrument.* (2017). Available online at: https://www.agreetrust.org/resource-centre/agree-ii (accessed February 10, 2026).

[17] Chen Y Yang K Marušic A Qaseem A Meerpohl JJ Flottorp S et al. A reporting tool for practice guidelines in health care: the right statement. *Ann Intern Med.* (2017) 166:128–32. 10.7326/M16-1565 27893062

[18] Lundeberg T Lund I Sing A Näslund J. Is placebo acupuncture what it is intended to be? *Evid Based Complement Alternat Med.* (2011) 2011:932407. 10.1093/ecam/nep049 19525330 PMC3139519

[19] Garner P Hopewell S Chandler J MacLehose H Schünemann HJ Akl EA et al. When and how to update systematic reviews: consensus and checklist. *BMJ.* (2016) 354:i3507. 10.1136/bmj.i3507 27443385 PMC4955793

[20] Jaeschke R Guyatt GH Dellinger P Schünemann H Levy MM Kunz R. GRADE working group. *BMJ.* (2008) 337:a744. 10.1136/bmj.a744 18669566

[21] Balagué F Mannion AF Pellisé F Cedraschi C. Non-specific LBP. *The Lancet.* (2012) 379:482–91. 10.1016/S0140-6736(11)60610-7 21982256

[22] Chou R Qaseem A Snow V Casey D Cross JT Shekelle P et al. Clinical efficacy assessment subcommittee of the American college of physicians, american college of physicians, American pain society Lbp guidelines panel. diagnosis and treatment of LBP: a joint clinical practice guideline from the american college of physicians and the american pain society. *Ann Intern Med.* (2007) 147:478–91. 10.7326/0003-4819-147-7-200710020-00006 17909209

[23] Foster NE Anema JR Cherkin D Chou R Cohen SP Gross DP et al. Lancet LBP series working group.prevention and treatment of LBP: evidence, challenges, and promising directions. *The Lancet.* (2018) 391:2368–83. 10.1016/S0140-6736(18)30489-6 29573872

[24] Nicol V Verdaguer C Daste C Bisseriex H Lefèvre-Colau MM. A narrative review of recent international guidelines for diagnosis and conservative treatment. (2023) 12:1685. 10.3390/jcm12041685 36836220 PMC9964474

[25] Kreiner DS Matz P Bono CM Cho CH Easa JE Ghiselli G. Guideline summary review: an evidence-based clinical guideline for the diagnosis and treatment of LBP. *Spine J.* (2020) 20:998–1024. 10.1016/j.spinee.2020.04.006 32333996

[26] Zhang D Fu Z Sun J Song Y Chiu P-E Chou L-W. Effects of Fu’s subcutaneous needling on clinical efficacy and psychological cognitive characteristics in patients with chronic non-specific LBP: a randomized controlled trial. *Complementary Ther Med.* (2024) 85:103080. 10.1016/j.ctim.2024.103080 39214379

[27] Coutaux A. Non-pharmacological treatments for pain relief: TENS and acupuncture. *Joint Bone Spine.* (2017) 84:657–61. 10.1016/j.jbspin.2017.02.005 28219657

[28] Giovanardi CM Gonzalez-Lorenzo M Poini A Marchi E Culcasi A Ursini F. Acupuncture as an alternative or in addition to conventional treatment for chronic non-specific LBP: a systematic review and meta-analysis. *Integrative Med Res.* (2023) 12:100972. 10.1016/j.imr.2023.100972 37637183 PMC10448023

[29] Zhang R Lao L Ren K Berman BM. Mechanisms of acupuncture–electroacupuncture on persistent pain. *Anesthesiology.* (2014) 120:482. 10.1097/ALN.0000000000000101 24322588 PMC3947586

[30] Mu J Furlan AD Lam WY Hsu MY Ning Z Lao L. Acupuncture for CNLBP. *Cochrane Database Syst Rev.* (2020) 12:CD013814. 10.1002/14651858.CD013814 33306198 PMC8095030

[31] Yao S Lu CJ Chen YL Zeng Z Li H. Methodology on development and revision for Chinese medicine (integrative medicine) clinical practice guidelines: The definition and classification of clinical practice guidelines. *China J Chinese Med.* (2016) 31:165–8.

[32] Ding N Wu XD Zhao NQ Mu DX Hu J Dong GF. Suggestions on the implementation of consensus method in the formulation of acupuncture-moxibustion clinical practice guidelines. *Chinese Acupuncture Moxibustion.* (2025) 45:237–41. 10.13703/j.0255-2930.20240718-0001 39943768

