# Supplementary material for: Protocol for a clinical practice guideline on acupuncture for chronic non-specific low back pain
Source: Front Med (Lausanne). 2026 Jul 6;13:1834549. doi: 10.3389/fmed.2026.1834549 (PMC13381246; doi:10.3389/fmed.2026.1834549)
Supplement: Supplementary file 1 [file Data_Sheet_1.pdf]

Supplementary material 2. Committee list

Guideline Steering Committee

1.Jingui Wang (The First Affiliated Hospital of Tianjin University of Traditional Chinese Medicine)
2.Huanan Li (The First Affiliated Hospital of Tianjin University of Traditional Chinese Medicine)
3.Jingbo Zhai (Tianjin University of Traditional Chinese Medicine)
4.Long Qu (Heilongjiang Academy of Traditional Chinese Medicine)
5.Juan Li (Chengdu University of Traditional Chinese Medicine)
6.Kunmu Zhang (The Second Affiliated Hospital of Fujian University of Traditional Chinese Medicine)
7.Bo Li (The First Affiliated Hospital of Tianjin University of Traditional Chinese Medicine)
8.Lili Zhang (The First Affiliated Hospital of Tianjin University of Traditional Chinese Medicine)
9.Xiang Zhou (Hangzhou Hospital of Traditional Chinese Medicine)

Guideline Consensus Expert Group

1.Zhiyong Fan (Guangdong Provincial Hospital of Traditional Chinese Medicine)
2.Wei Fang (The First Affiliated Hospital of Tianjin University of Traditional Chinese Medicine)
3.Yufeng He (The First Affiliated Hospital of Guangxi University of Traditional Chinese Medicine)
4.Yinghui Jin (Zhongnan Hospital of Wuhan University)
5.Bo Li (The First Affiliated Hospital of Tianjin University of Traditional Chinese Medicine)
6.Jianhua Li (Yueyang Hospital of Integrated Traditional Chinese and Western Medicine, Shanghai University of Traditional Chinese Medicine)
7.Juan Li (Chengdu University of Traditional Chinese Medicine)
8.Yikai Li (Southern Medical University)
9.Aifeng Liu (The First Affiliated Hospital of Tianjin University of Traditional Chinese Medicine)
10.Hongliang Tang (Fangchenggang Hospital Affiliated to Guangxi University of Traditional Chinese Medicine)
11.Weigang Wang (The Affiliated Hospital of Shaanxi University of Traditional Chinese Medicine)
12.Yufeng Wang (Changchun University of Traditional Chinese Medicine)

13.Yizhou Wang (The Affiliated Hospital of Tianjin Academy of Traditional Chinese Medicine Research)
14.Yunchuan Wu (Nanjing University of Traditional Chinese Medicine)
15.Changhe Yu (Dongzhimen Hospital of Beijing University of Traditional Chinese Medicine)
16.Yong Ye (The First Affiliated Hospital of Hunan University of Traditional Chinese Medicine)
17.Wei Zhang (The First Affiliated Hospital of Tianjin University of Traditional Chinese Medicine)

Guideline secretarial group
1.An Bao (The First Affiliated Hospital of Tianjin University of Traditional Chinese Medicine)
2.Yingying Chen (The First Affiliated Hospital of Tianjin University of Traditional Chinese Medicine)
3.Qiujun Wu (The First Affiliated Hospital of Tianjin University of Traditional Chinese Medicine)
4.Jinyang Li (The First Affiliated Hospital of Tianjin University of Traditional Chinese Medicine)
5.Weiran Shang (The First Affiliated Hospital of Tianjin University of Traditional Chinese Medicine)

Guideline evidence evaluation group
1.Jiacheng Zhang (The First Affiliated Hospital of Tianjin University of Traditional Chinese Medicine)
2.Gaoxinli Liu (Baoding Hospital of Guang'anmen Hospital)
3.Shun Fan (The First Affiliated Hospital of Tianjin University of Traditional Chinese Medicine)
4.Yusheng Li (The First Affiliated Hospital of Tianjin University of Traditional Chinese Medicine)
5.Jing Ning (Jiangxi University of Traditional Chinese Medicine)
6.Xiaoyu Wang (Guangdong Provincial Hospital of Traditional Chinese Medicine)

Treatment Protocol Development Group
1.Jinshan Bai (Pinggu District Hospital of Traditional Chinese Medicine, Beijing)
2.Shaotao Chen (Changchun University of Traditional Chinese Medicine)
3.Honggen Du (The First Affiliated Hospital of Zhejiang University of Traditional Chinese Medicine)

4.Hongyuan Fan (The First Affiliated Hospital of Guizhou University of Traditional Chinese Medicine)
5.Zhiyong Fan (Guangdong Provincial Hospital of Traditional Chinese Medicine)
6.Ruiyang Fu (Huzhou Hospital of Traditional Chinese Medicine)
7.Meng Guo (The Third Clinical Hospital Affiliated to Changchun University of Traditional Chinese Medicine)
8.Rusong Guo (Guangdong Provincial Hospital of Traditional Chinese Medicine)
9.Sheng Guo (Oriental Hospital of Beijing University of Traditional Chinese Medicine)
10.Yufeng He (The First Affiliated Hospital of Guangxi University of Traditional Chinese Medicine)
11.Jianhua Li (Yueyang Hospital of Integrated Traditional Chinese and Western Medicine, Shanghai University of Traditional Chinese Medicine)
12.Jinlong Li (Hebei University of Traditional Chinese Medicine)
13.Keyi Li (Ruikang Hospital Affiliated to Guangxi University of Traditional Chinese Medicine)
14.Yikai Li (Southern Medical University)
15.Qiang Lyu (Shuguang Hospital Affiliated to Shanghai University of Traditional Chinese Medicine)
16.Qi Tao (Jiangsu Provincial Hospital of Traditional Chinese Medicine)
17.Hongliang Tang (Fangchenggang Hospital Affiliated to Guangxi University of Traditional Chinese Medicine)
18.Weigang Wang (The Affiliated Hospital of Shaanxi University of Traditional Chinese Medicine)
19.Yufeng Wang (Changchun University of Traditional Chinese Medicine)
20.Yizhou Wang (The Affiliated Hospital of Tianjin Academy of Traditional Chinese Medicine Research)
21.Jianmin Wu (Gansu University of Traditional Chinese Medicine)
22.Yunchuan Wu (Nanjing University of Traditional Chinese Medicine)
23.Weiguo Xue (Beijing University of Traditional Chinese Medicine)
24.Aiguo Yang (The Affiliated Hospital of Chengdu University)
26.Yong Ye (The First Affiliated Hospital of Hunan University of Traditional Chinese Medicine)
26.Jiajun You (Ninghai Hospital of Traditional Chinese Medicine)
27.Zhen Yan (Yueyang Hospital of Integrated Traditional Chinese and Western Medicine, Shanghai University of Traditional Chinese Medicine)
28.Lizhi Zhang (Shandong Provincial Hospital for Chronic Diseases)

29.Shiqing Zhang (The First Affiliated Hospital of Henan University of Traditional Chinese Medicine)
30.Xin Zhang (Changchun University of Traditional Chinese Medicine)
31.Bin Zhou (The Third Affiliated Hospital of Henan University of Traditional Chinese Medicine)
32.Zhiyue Zhou (Liaoyuan Hospital of Traditional Chinese Medicine)
33.Qingguang Zhu (Yueyang Hospital of Integrated Traditional Chinese and Western Medicine, Shanghai University of Traditional Chinese Medicine)

Guideline external review group

1.Ling Li (West China Hospital, Sichuan University)
2.Jiaguo Zhao (Beijing Tongren Hospital)
