# Supplementary material for: Protocol for a clinical practice guideline on acupuncture for chronic non-specific low back pain
Source: Front Med (Lausanne). 2026 Jul 6;13:1834549. doi: 10.3389/fmed.2026.1834549 (PMC13381246; doi:10.3389/fmed.2026.1834549)
Supplement: Supplementary file 2 [file Data_Sheet_2.pdf]

Supplementary material 2 .Search Strategies

Chinese Search Strategies

题名或关键词:(下腰痛 OR 腰背痛 OR 腰痛 OR 腰痹 OR 腰腿痛 OR 非特异性下腰痛 OR 慢性非特异性下腰痛 OR 腰肌劳损 OR 第三腰椎横突综合症 OR 腰背肌筋膜炎 OR 腰肌筋膜炎 OR 腰臀肌筋膜炎 OR 腰臀肌筋膜疼痛综合症) AND 题名或关键词：(随机 OR 试验 OR 系统评价 OR 观察 OR 疗效 OR Meta OR 指南 OR 共识) AND 题名或关键词：(针灸 OR 针刺 OR 电针 OR 火针 OR 耳针 OR 毫针 OR 三棱针)

English Search Strategies

#1 "Acupuncture Therapy"[MeSH Terms] OR "Acupuncture, ear"[MeSH Terms] OR "Acupuncture Points"[MeSH Terms] OR "Acupuncture"[MeSH Terms] OR " Acupuncture Analgesia"[MeSH Terms] OR "Electroacupuncture"[MeSH Terms]

#2 "Acupuncture Therapy"[Title/Abstract] OR "Acupuncture, Ear"[Title/Abstract] OR "Acupuncture Point*"[Title/Abstract] OR "Acupuncture"[Title/Abstract] OR "Acupuncture Analgesia"[Title/Abstract] OR "Electroacupuncture"[Title/Abstract] OR "Acupuncture Treatment*"[Title/Abstract] OR "Treatment, Acupuncture"[Title/Abstract] OR "Therapy, Acupuncture"[Title/Abstract] OR "Acupotomy"[Title/Abstract] OR "Acupotomies"[Title/Abstract] OR "Ear Acupuncture*"[Title/Abstract] OR "Acupuncture*, Auricular"[Title/Abstract] OR "Auricular Acupuncture*"[Title/Abstract] OR "Point*, Acupuncture"[Title/Abstract] OR "Acupoint*"[Title/Abstract] OR "Analgesia, Acupuncture"[Title/Abstract] OR "Acupuncture Anesthesia"[Title/Abstract] OR "Anesthesia, Acupuncture"[Title/Abstract]

#3 #1 OR #2

#4 "Low Back Pain"[MeSH Terms]

#5 "Low Back Pain"[Title/Abstract] OR "Back Pain*, Low"[Title/Abstract] OR "Pain*, Low Back"[Title/Abstract] OR "Back Ache*, Low"[Title/Abstract] OR "Ache*, Low Back"[Title/Abstract] OR "Low Back Ache*"[Title/Abstract] OR "Low Backache*"[Title/Abstract] OR "Backache*, Low"[Title/Abstract] OR "Lower Back Pain*"[Title/Abstract] OR "Pain*, Lower Back"[Title/Abstract] OR "Back Pain*, Lower"[Title/Abstract] OR "Lumbago"[Title/Abstract] OR "Low Back Pain, Mechanical"[Title/Abstract] OR "Mechanical Low Back Pain"[Title/Abstract] OR "Low Back Pain, Posterior Compartment"[Title/Abstract] OR "Low Back Pain, Postural"[Title/Abstract] OR "Postural Low Back Pain"[Title/Abstract] OR "Low Back Pain, Recurrent"[Title/Abstract] OR "Recurrent Low Back Pain"[Title/Abstract] OR "Nonspecific Low Back Pain"[Title/Abstract] OR "Chronic Nonspecific Low Back Pain"[Title/Abstract] OR "Lumbodynia"[Title/Abstract] OR "Lumbar Pain"[Title/Abstract] OR "Osphyalgia"[Title/Abstract] OR "Lumbar Myalgia"[Title/Abstract] OR "Third Lumbar Transverse Process

syndrome"[Title/Abstract] OR "Lumbar Muscle Fasciitis"[Title/Abstract] OR "Lumbar Gluteal Myofascitis"[Title/Abstract] OR "NLBP"[Title/Abstract] OR "CNLBP"[Title/Abstract] OR "NSLBP"[Title/Abstract] OR "CNSLBP"[Title/Abstract]

#6 #4 OR #5

#7 ("Clinical"[Title/Abstract] AND "Trial"[Title/Abstract]) OR "Clinical trials as topic"[MeSH Terms] OR "Clinical trial"[Publication Type] OR Random*[Title/Abstract] OR Random Allocation[MeSH Terms] OR "Meta-Analysis"[Title/Abstract] OR "Systematic Review"[Title/Abstract] OR "Guideline"[Title/Abstract] OR "Consensus"[Title/Abstract]

#8 #3 AND #6 AND #7
