# Supplementary material for: Protocol for a clinical practice guideline on acupuncture for chronic non-specific low back pain
Source: Front Med (Lausanne). 2026 Jul 6;13:1834549. doi: 10.3389/fmed.2026.1834549 (PMC13381246; doi:10.3389/fmed.2026.1834549)
Supplement: Supplementary file 3 [file Data_Sheet_3.pdf]

Supplementary material 3 .Selection and Engagement of Patient Representatives

Inclusion Criteria

1.Meet the diagnostic criteria for chronic nonspecific low back pain (CNLBP).
2.Aged 18 years or older.
3.Have lived experience of chronic nonspecific low back pain, can represent the views of a broad patient population, and do not participate solely for academic or professional interests. Priority is given to those who have received acupuncture treatment.
4.Have good communication and teamwork skills, and can actively express their views during consensus discussions.
5.Can attend all scheduled consensus meetings and complete relevant voting tasks.
6.Have no severe mental disorders or cognitive impairments that may affect effective participation.

Recruitment Methods

Patient representatives for this guideline will be publicly recruited from the outpatient department of the First Teaching Hospital of Tianjin University of Traditional Chinese Medicine and local communities. Applicants will be selected through completing an application form and attending an informal interview. All patient representatives must sign an informed consent form and a conflict of interest declaration form. Conflicts of interest will be assessed by the Guideline Steering Committee. Before the consensus meetings, the Secretariat Group will organize specialized training for patient representatives. The training will cover an introduction to CNLBP, basic knowledge of acupuncture, the guideline development process, as well as a systematic explanation of clinical questions, outcome indicators, GRADE evidence grading, and methods for formulating recommendations. It will also help them become familiar with voting procedures and understand the significance of their participation in developing this guideline.

Specific Responsibilities of Patient Representatives

1.Assess and vote on the importance of clinical questions and outcome indicators from the patient perspective. Their votes on clinical questions and outcome indicators will have equal weight as those of the Guideline Consensus Expert Group members, and together they will determine the final ranking of clinical questions and outcome indicators.
2.Participate in discussions on clinical questions and outcome indicators during consensus meetings and express their views based on personal treatment experience.
3.Review the draft guideline manuscript and provide feedback on the clarity, understandability, and clinical applicability of recommendations and treatment protocols.

The Guideline Consensus Expert Group and the Secretariat Group will provide professional explanations of guideline content to patient representatives throughout the process. All feedback from patient representatives will be fully documented and archived by the Secretariat Group and considered during the guideline development process.
